# The Novel and Minimally Invasive Treatment Modalities for Female Pelvic Floor Muscle Dysfunction; Beyond the Traditional

**DOI:** 10.4274/balkanmedj.2018.0869

**Published:** 2018-09-21

**Authors:** Yiğit Akın, Matthew Young, Muhammad Elmussareh, Nickolaus Charalampogiannis, Ali Serdar Gözen

**Affiliations:** 1 Department of Urology, İzmir Katip Çelebi University School of Medicine, İzmir, Turkey; 2 Clinic of Urology, Mid Yorkshire Hospitals NHS Trust, Wakefield, The United Kingdom; 3 Department of Urology, SLK-Kliniken Heilbronn, University of Heidelberg, Heilbronn, Germany

**Keywords:** Minimally invasive surgery, pelvic floor, pelvic organ prolapse, stress urinary incontinence

## Abstract

Pelvic floor dysfunction is a clinical entity that is prevalent among female patients. Determining the exact underlying cause of pelvic floor dysfunction is difficult, and surgical intervention for this clinical entity may be challenging. Pelvic floor dysfunction can affect the quality of life of the patient by causing stress urinary incontinence, pelvic organ prolapse, or both. Well-defined surgical treatment options, minimally invasive approaches, and novel techniques for the treatment of pelvic floor dysfunction have been recently introduced. Here, we evaluated the management options available for patients with stress urinary incontinence and pelvic organ prolapse. We searched Medline and EMBASE databases for relevant articles by using the keywords “pelvic floor dysfunction,” “minimally invasive procedures,” “stress urinary incontinence,” “pelvic organ prolapse,” and “novel techniques”. Traditional treatment options for stress urinary incontinence and pelvic organ prolapse are beyond the scope of our review. Laparoscopic and robotic surgical treatments for pelvic floor dysfunction continue to evolve and develop. These minimally invasive techniques will soon replace open procedures. Alternative novel treatment modalities have also been developed from novel human-compatible materials and are emerging as successful treatments for stress urinary incontinence. The development of these various treatment options has implications for future surgical practice in the field of uro-gynecology.

The pelvic floor, which comprises pelvic bone, muscles and connective tissue, supports and is vital for the normal functions of the pelvic organs, particularly the urinary bladder, urethra, rectum, and the reproductive system (1,2). Pelvic floor dysfunction (PFD) is a collection of complex clinical findings. The symptoms of PFD include pelvic pain, pressure, dyspareunia, stress urinary incontinence (SUI), incomplete urinary voiding, defecatory dysfunction, and pelvic organ prolapse (POP) (3). PFD is more common among females than among males and is often the result of vaginal childbirth (4,5). The other recognized major risk factors for PFD include age, obesity, menopause, and pregnancy (5,6). PFD affects the quality of life of patients and therefore is of great clinical importance (7). Surgical intervention remains the definitive treatment option for patients with symptomatic POP or SUI. In a large population-based study in the United States, the lifetime risk of any primary surgery for SUI or POP reaches 20% ages of 80 years women (8). The American Urology Association quotes a high percentage of 30% in patients undergoing surgery for SUI (9). Surgical and medical technologies in this field have rapidly advanced over the past two decades (10). Minimally invasive surgical options available to patients have expanded, and alternative and novel approaches, such as biological tissue fibrin materials and injectable biological agents (IBAs), have been developed to improve the management of SUI and POP secondary to PFD. Given the increased prevalence of obesity among the aging general population, PFD will become an ever-increasing presentation to uro-gynecologists and specialists of female urology. Minimally invasive treatments are therefore vital to improve the management of this growing cohort of patients.

Here, we describe the clinical presentation and assessment of patients presenting with SUI and POP and summarize the evidence for various alternative and minimally invasive approaches for PFD treatment. We have avoided focusing on traditional conservative treatments, namely pelvic floor exercises and the widely accepted practice of midurethral tape surgery, because these treatment modalities are well reported in current literature on PFD management.

## STRESS URINARY INCONTINENCE

SUI is described as “the observation of involuntary leakage from the urethra, synchronous with exertion/effort, or on sneezing or coughing” (11). The prevalence of SUI increases among the female population with age. For example, the prevalence of SUI increases from 16% among women under 30 years to 29% among women aged 30-60 years old (12). Well-documented risk factors for the development of SUI include childbirth, childbirth mode, obesity, smoking, and age (13,14,15,16). Cesarean sections, a mode of childbirth, exert a protective effect on the pelvic floor reported by Al-Mufti et al. (17). Alternative differential diagnoses must be considered prior to embarking on treatment for SUI. Patients may also experience the symptoms of urge urinary incontinence (UUI) in addition to SUI. The clinician must correctly identify the predominant symptoms to provide the most appropriate treatment to patients with mixed urinary incontinence. UUI is often treated medically upon the first occurrence, whereas SUI often requires additional intervention (18). In addition to providing their clinical history, patients should also complete a bladder diary to assess their fluid intake and voiding habits, as well as the frequency of incontinence episodes. This assessment should be followed by a detailed physical examination, including neurological assessment (19). Pad tests and Q-tip tests can be useful in determining whether the patient will benefit from a urethral sling. Urodynamic studies can help confirm the patients’ diagnosis, particularly prior to surgical intervention (20). Conservative options, mainly pelvic floor exercises and weight loss, are usually the first line of treatment for SUI. Other nonsurgical options include electrical and magnetic stimulation, duloxetine use, vaginal inserts (incontinence pessaries and tampons), and topical estrogens (21,22,23). Despite these treatments, as many as 30% of patients with SUI undergo surgical interventions (9). Various surgical techniques for SUI exist. These techniques include Burch retropubic colposuspension, tension-free vaginal tapes, transobturator tapes, midurethral slings, and mini-slings (24). Sling operations often require the use of prolene mesh devices (25). The use of paravaginal grafting techniques has also been reported (26). The increased scrutiny and additional restrictions received by the use of mesh devices (27) in the last decade highlight the need for novel and alternative treatments for the management of POP and SUI.

### Stress Urinary Incontinence Treatments

### Radiofrequency Denaturation

Radiofrequency denaturation is a nonsurgical technique that involves the insertion of a device into the urethra under local anesthesia. Radiofrequency energy is then applied to the bladder neck and proximal urethra to denature and promote the remodeling of collagen in the surrounding tissue (28). Patient outcomes were variable with reported “cure rates” of 22%-67% (29). A 3-year prospective study showed significant improvement in the patients’ quality of life following treatment with RD but did not compare RD with other treatments (30).

A Cochrane systematic review concluded that insufficient evidence exists to determine whether RD improves the symptoms of SUI when compared with the sham treatment (31). 

No evidence supports that RD is comparable with other established treatments for SUI, such as pelvic floor physiotherapy, pessaries, surgery, or IBAs. The recurrence of lower urinary tract symptoms within 3 years of treatment delivery has been reported, with dysuria being the most common complaint (29). Additional randomized controlled trials are needed to accurately determine the efficacy of RD in clinical practice. 

### Injectable Biological Agents

IBAs have been used for several decades. These materials are applied to increase tissue volume within the proximal urethral wall between the bladder neck and the external urethral sphincter. Increasing tissue volume at these locations increases urethral luminal coaptation and bladder outflow resistance (32). IBAs are delivered endoscopically with a cystoscope via needle injection into the periurethral area. The European Association of Urology guidelines recommend the use of IBAs for the temporary treatment of symptoms in patients who have failed conservative treatments for SUI. It can also be offered an alternative to a midurethral sling (33).

Various bulking agents have been developed and trialed. These IBAs include autologous fat, cross-linked collagen, graphite-coated zirconium beads, polytetrafluroethylene, silicon, dimethylsulfoxide and ethylene vinyl alcohol copolymers, hyaluronic acid, dextranomer microspheres, and calcium hydroxyapatite (34,35,36). Treatment with IBAs improves SUI symptoms by 18%-40% (28). The efficacy of IBAs is superior to that of pelvic floor physiotherapy but is inferior to that of surgical management (36). Collagen has been removed from the clinical arena but has been used as the standard reference for new agents in clinical trials (35). A Cochrane systematic review found that none of the new agents are inferior to collagen but have failed to reach a consensus on the superior agent or the effect of injection location within the urethra on patient outcomes (35). Novel IBAs (polyacrylamide hydrogel) have decreased patient incontinence episodes by 50% or greater in 53.2% of 12 months after treatment (37). IBAs may be cost effective in the initial treatment of patients with SUI without hypermobility or as a surgical adjunct. However, their long-term (greater than 15 months) economic viability is questionable when compared with that of traditional sling surgery for SUI (35). Common complications following the injection of IBAs include urinary retention (up to 30%) and urinary tract infection (up to 25%) (38). Rare complications include abscess formation following collagen injection and fat embolism after autologous fat injection (38). 

### Stem-cell Injections for Urethral Sphincter Restoration

This treatment aims to restore the external urethral sphincter through the injection of stem cells (often skeletal muscle-derived or adipose tissue-derived) into and around the sphincter (39). This treatment has been developed in animal models by Xu et al. (40), who successfully demonstrated the restoration of the urethral sphincter in a pudendal nerve-transected rat following the injection of muscle-based stem cells. Recently, a small phase-one clinical trial on the outcomes of the periurethral injection of stem cells has been reported. Arjmand et al. (41) reported favorable outcomes for women treated with autologous adipose-derived stem cells injected into the periurethral area for SUI (42). Core blood stem cells have been used by Lee et al. (42) with reasonable success (n=39) in female patients with SUI. Among these patients, 67% showed improvement at 12 months postinjection. Peters et al. (43) reported favorable outcomes following the injection of increasing doses of autologous muscle-derived stem cells into the urinary sphincter. However, several publications reported minimal improvement in voiding or in the results of urodynamic assessment, as well as the delayed onset of symptom improvement (44,45). This treatment modality remains in its infancy, with evidence to date being collected mainly from animal models and small-scale phase-one clinical trials. Ethical considerations and concerns regarding the regulatory control of stem-cell research have affected the expansion of this field (45).

### Fibrin Sealant

Biocompatible fibrin glue is another endoscopic treatment for SUI that has existed since the 1990s (46,47). It involves the transvaginal placement of fibrin sealant to stimulate a fibrotic reaction, which elevates the vesicle–urethral junction. Data on the long-term outcomes of this treatment option remain lacking, with few published articles since the late 1990s. 

### Laparoscopic and Robotic-assisted Surgical Modalities

Open Burch colposuspension was the gold standard surgical technique for the management of SUI until the early 1990s (48). At 1 year postoperation, 85%-90% of patients are continent. This rate drops to 70% at 5 years postoperation. In 1991, Vancaillie and Schuessler (49) successfully reported the first laparoscopic Burch colposuspension. Following its introduction into clinical practice, laparoscopic treatment for SUI has become increasingly adopted, and evidence showing that its clinical outcomes are equivalent to that of colposuspension with the added benefits of minimally invasive surgery has accumulated. These benefits include reduced blood loss, length of hospital stay, postoperative pain, and catheterization period (50,51,52). Some authors have argued that laparoscopic colposuspension should be considered as the treatment of choice for women, especially young women, undergoing pelvic floor repair and concomitant retropubic surgery because it avoids the well-documented complications of mesh migration and erosion (53). Laparoscopic techniques for colposuspension using mesh and staples instead than the classical suturing technique have been described. A randomized controlled trial, however, has shown that this technique is associated with unfavorable outcomes (54). The challenging and most time-consuming aspect of laparoscopic colposuspension is the process of laparoscopic suturing in the pelvis. The development of robotic surgical systems has attempted to overcome this challenge (55). Robotic systems have revolutionized pelvic surgery, particularly uro-pelvic oncology. Three-dimensional-image displays and 720-degree robotic arm articulation have considerably facilitated suture-intensive procedures, such as laparoscopic colposuspension. Successful feasibility studies on the role of robotic-assisted surgery in SUI and voiding dysfunction after urogynaecological surgery have been conducted over the last 3-4 years (56,57). Modified single-series robotic-assisted approaches have been described in the contemporary literature with successful outcomes (58). No study has compared the outcomes of robotic-assisted techniques with either open or laparoscopic colposuspension. If benign uro-gynecological surgery follows the same trend as other pelvic surgical specialties, then robotic-assisted surgery for SUI is likely to become an increasing popular and cost effective technique in this specialty. SUI treatment modalities based on minimally invasive and laparoscopic and robotic interventions are summarized in Table 1. The published literature on novel techniques for the management of patients with SUI is presented in Table 2.

## PELVIC ORGAN PROLAPSE

POP in females can be defined as the descent and/or herniation of pelvic organs from their normal anatomical location toward or through the vaginal opening. This condition can affect the patient’s quality of life and sexual function (59). In females, the utero-sacral ligament, paravaginal attachments, and perineal body constitute the main parts of the system that supports pelvic organs and are interconnected with the endopelvic fascia (60). Any defect in this network may cause POP. Sacral nerve roots S2-4, via the pudendal nerve, are vitally important in the function of the pelvic floor. Defects in neurological communication in these nerves can interfere with the integrity of the pelvic organs and the function of the pelvic floor. Risk factors for the development of POP are similar to those for the development of SUI. Aging, multiparity, and obesity increase the prevalence of POP (61,62,63). Previous hysterectomy is also a risk factor for POP (63). Chronic constipation and ethnicity (Caucasian, followed by Latin-American, followed by Africa-American women in decreasing order of prevalence) have also been implicated in the development of POP (64,65). Many patients with POP are asymptomatic. However, symptomatic patients can present with a variety of symptoms that may be specifically related to prolapsed structures, such as a bulge or the sensation of pressure within the vagina. Other symptoms include lower urinary tractand defecatory or sexual dysfunction symptoms (63). POP and SUI symptoms considerably overlap (66). As with SUI, the patient’s complete medical history must be collected and a thorough physical examination must be performed as part of the initial assessment of POP. POP is classified into four levels in accordance with the descriptions provided by The International Continence Society (67): 

Level 1: Distal part of POP is up to 1 cm over the hymen.

Level 2: Distal part of POP is 1 cm or more over the hymen.

Level 3: Distal part of POP exceeds 1cm over the hymen and is less than 2 cm outside the body.

Level 4: Complete vaginal eversion.

Treatment options can be broadly divided into conservative or surgical options. Conservative measures include smoking cessation and lifestyle modifications (increased exercise, weight loss, and pelvic floor exercises) (68). Vaginal pessaries are widely used to successfully control symptoms with success rates of 50%-70% (69,70). Surgical treatments are offered to patients who have declined or failed conservative measures. 

Most women with symptomatic POP that continues to persist despite conservative measures are treated through reconstructive procedures. Obliterated procedures are reserved for women who cannot tolerate major surgery or who are not sexually active.

### Pelvic Organ Prolapse Treatment Options

### Transvaginal Sacrospinous Ligament Suspension Stapled Fixation

In this surgical technique, the bilateral sacrospinous ligament is suspended by using surgical staples. It was first described in 1997 by Febbraro et al. (71) in a case series of 34 patients with levels 3 or 4 POP. The sacrospinous ligament suture fixation is a well-documented surgical treatment for POP, with acceptable complication rates and cure rates of 50%-100% (72). The cost of stapling devices is the limiting step in the technique described by Febbraro et al. (71) and when compared with a cheaper and equally effective existing technique, the stapled method is not cost effective. 

### Anterior Suturing Device

The use of a suturing device (Capio^®^) for the fixation of the sacrospinous ligament has been recently described. The device is a suture-performing system with a taper-cut needle and attached suture. The needle carrier is enclosed in the concave distal segment of the device’s shaft. It is designed to allow the surgeon to drive and retrieve the suture in one step. In a comparative case series, Leone Roberti Maggiore et al. (73) found that traditional sutured fixation using the Capio^®^ system reduced operative time and reduce blood loss while delivering comparable clinical outcomes at 3-year follow-up (73). Other observational series have also reported favorable outcomes, with reported cure rates of nearly 90% and only 10.6% POP recurrence (74).

### Laparoscopic Sacrocolpopexy

Laparoscopic sacrocolpopexy was introduced in 1991 as an adaptation of the well-described open surgical approach. Open sacrocolpopexy was widely regarded as the gold-standard surgical treatment for POP with long-term success rates of 78%-100% (75). A randomized controlled trial by Freeman et al. (76) revealed clinical equivalence between open and laparoscopic sacrocolpopexy. Numerous retrospective case series have confirmed that the laparoscopic approach is a safe and effective alternative treatment for the surgical management of POP while conferring the well-documented advantages of minimally invasive surgery. Laparoscopic surgery has been shown to reduce surgical complications (7.7% for open and 4% for laparoscopic repair), pain, UTI rate, and urinary retention (75). Although reoperation rates for POP were higher in the laparoscopic group (5.7%) than in the open surgery group (3.8%), this difference was not statistically significant (p=0.29) (75). Similar findings have been reported by other authors (77,78) with excellent 5-year anatomical and functional outcomes being reported by Sarlos al. (79). As with any laparoscopic pelvic procedure, the main learning curve for the procedure centers on the mastery of laparoscopic suturing in the tight confines of the pelvis (80). As robotic-assisted surgery gathers momentum to address this challenge, the role of the “traditional” laparoscopic sacrocolpopexy may become limited. 

### Robotic-assisted Laparoscopic Sacrocolpopexy

**Figure 1:**
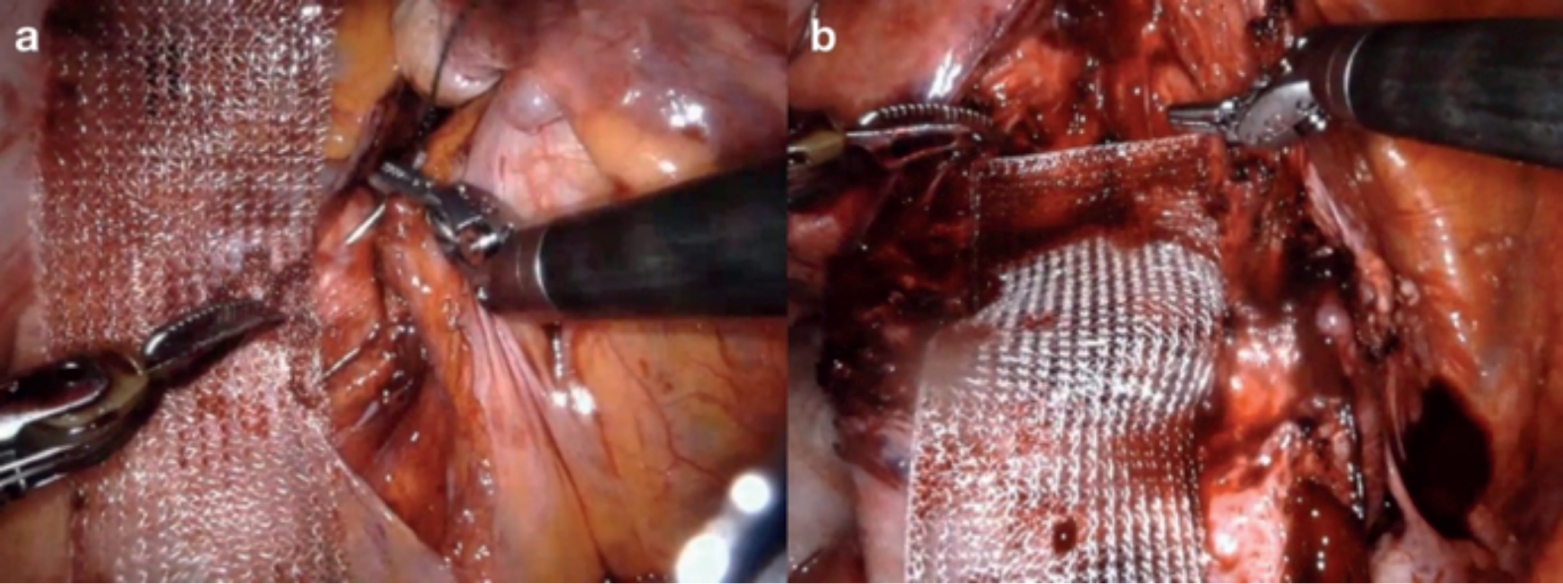
a,b. Dissection (a) and mesh graft placement (b) during robotic-assisted laparoscopic sacrocolpopexy.

**Figure 2:**
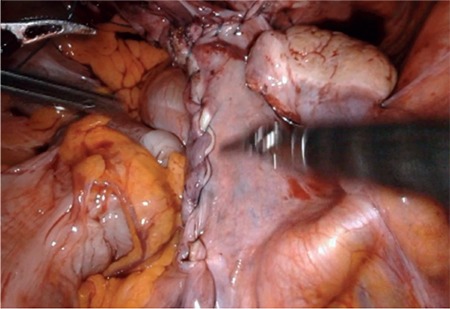
The mesh is placed under the peritoneum. All tissues are enclosed after robotic-assisted laparoscopic sacrocolpopexy.

The ever-expanding role of robotic-assisted procedures now includes sacrocolpopexy. The advantages of robotic-assisted pelvic procedures have been extensively reported in the literature (67,79). Robotic-assisted laparoscopic sacrocolpopexy (RAS) aims to overcome the lengthy learning curve associated with complex laparoscopic surgery. A large systematic review and meta-analysis of the published literature by Serati et al. (80) revealed that RAS is a safe and effective treatment option for patients with POP. When compared with open sacrocolpopexy, RAS increases operative time, but significantly reduces blood loss and length of hospital stay. Objective cure rates for RAS range from 84%-100% with the prolapse recurrence rate of 6.4% and reoperation rate of 3.3% (80). Interestingly, several articles have reported an overall cost benefit of RAS over open surgery. Figure 1 shows the dissection of tissues and placement of the mesh graft. Given that the mesh should not touch the bowel and intestine, surrounding tissues should be enclosed (Figure 2). A randomized controlled trial comparing RAS and open sacrocolpopexy showed that RAS does not significantly increase costs when the initial robot purchase and maintenance cost are excluded. Compared with laparoscopic sacrocolpopexy, RAS is associated with decreased blood loss and increased operative times (80). No significant difference exists between the clinical outcomes of RAS and laparoscopic sacrocolpopexy (80). POP treatment modalities based on minimally invasive and laparoscopic and robotic interventions are shown in Table 3. The published literature is summarized in Table 4. An awareness of alterative and novel treatments for PFD is crucial given the current controversy surrounding the use of mesh technology in uro-gynecological practice. Robotic-assisted surgery for SUI and POP is undergoing exponential development. The ability to offer a plethora of minimally invasive and nonsurgical techniques for the treatment of PFD has become increasingly necessary as the human population continues to age and individuals present with increasingly multiple medical comorbidities. 

## Figures and Tables

**Table 1:**
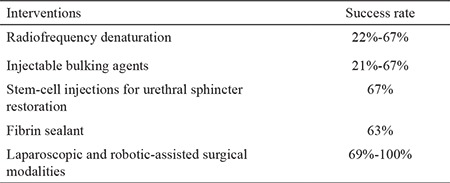
Stress urinary incontinence treatments based on minimally invasive and laparoscopic and robotic interventions

**Table 2:**
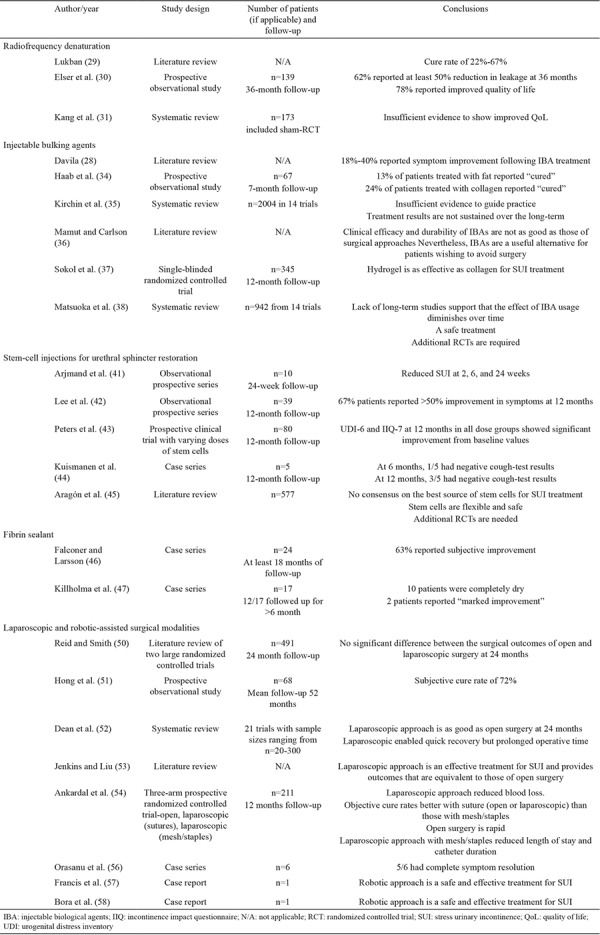
Summary of the literature reviewed related to novel techniques for the management of stress urinary incontinence

**Table 3:**
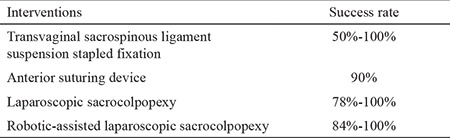
Pelvic organ prolapse treatment modalities based on minimally invasive and laparoscopic and robotic interventions

**Table 4:**
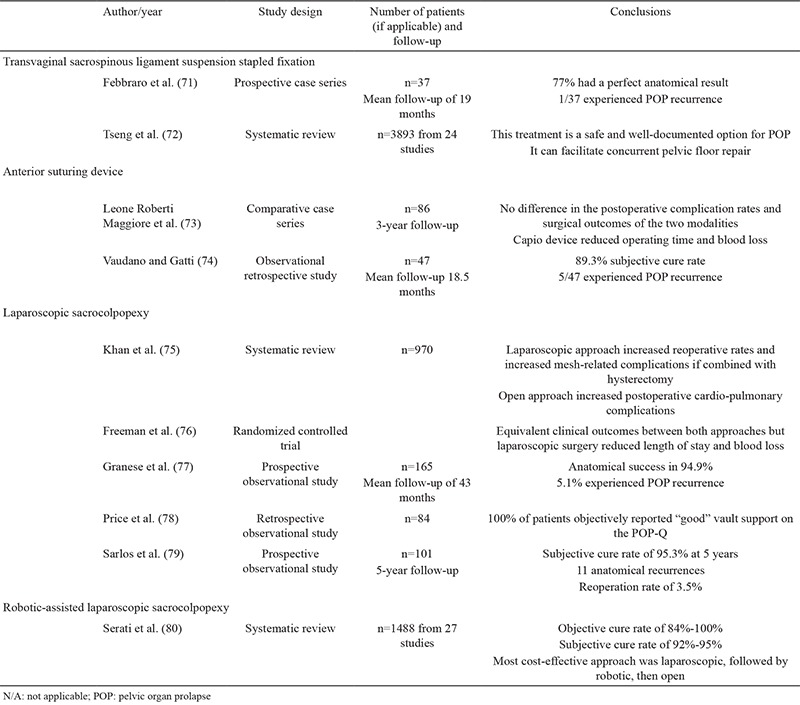
Summary of the literature reviewed related to novel techniques for the management of patients with pelvic organ prolapse
